# Complementary App-Based Yoga Home Exercise Therapy for Patients With Axial Spondyloarthritis: Usability Study

**DOI:** 10.2196/57185

**Published:** 2024-09-19

**Authors:** Lara Grube, Pascal Petit, Nicolas Vuillerme, Marlies Nitschke, Obioma Bertrand Nwosu, Johannes Knitza, Martin Krusche, Ann-Kristin Seifer, Bjoern M Eskofier, Georg Schett, Harriet Morf

**Affiliations:** 1 Department of Internal Medicine 3- Rheumatology & Immunology Universitätsklinikum Erlangen Friedrich-Alexander-Universität Erlangen-Nürnberg Erlangen Germany; 2 Deutsches Zentrum Immuntherapie Universitätsklinikum Erlangen Friedrich-Alexander-Universität Erlangen-Nürnberg Erlangen Germany; 3 AGEIS Université Grenoble Alpes Grenoble France; 4 Institut Universitaire de France Paris France; 5 LabCom Telecom4Health Orange Labs & Université Grenoble Alpes, CNRS, Inria Grenoble France; 6 Machine Learning and Data Analytics Lab Department Artificial Intelligence in Biomedical Engineering (AIBE) Friedrich-Alexander-Universität Erlangen-Nürnberg Erlangen Germany; 7 Institute for Digital Medicine University Hospital of Giessen and Marburg Philipps-University Marburg Marburg Germany; 8 III. Department of Medicine University Medical Center Hamburg-Eppendorf Hamburg Germany; 9 Translational Digital Health Group, Institute of AI for Health German Research Center for Environmental Health Helmholtz Zentrum München Neuherberg Germany

**Keywords:** DTx, DHA, usability, Yoga, YogiTherapy, ankylosing spondylitis, axial spondylarthritis, digital health application, eHealth, self-assessment, physical exercise, patient acceptance, therapy, home exercise, exercise, patients, patient, spondyloarthritis, usability study, app, apps, rheumatic disease, chronic, spine, adjacent joints, joints, joint, correlation analysis, digital therapeutics

## Abstract

**Background:**

Axial spondyloarthritis (AS) is a chronic inflammatory rheumatic disease characterized by potentially disabling inflammation of the spine and adjacent joints. Regular exercise is a cornerstone of treatment. However, patients with AS currently have little support. YogiTherapy (MaD Lab) is an app developed to support patients with AS by providing instructions for yoga-based home exercise therapy.

**Objective:**

This study aimed to evaluate the usability and acceptance of the newly designed YogiTherapy app for patients with AS.

**Methods:**

Patients completed the User Version of the Mobile Application Rating Scale (uMARS) and net promoter score (NPS) questionnaires after the app introduction. Wilcoxon Mann-Whitney rank sum test, chi-square test for count data, and correlation analysis were conducted to examine the usability of the app, acceptance, and patient characteristics.

**Results:**

A total of 65 patients with AS (33, 51% female; age: mean 43.3, SD 13.6 years) were included in the study from May 2022 to June 2023. Subsequently, the data were analyzed. Usability was rated moderate, with a mean uMARS of 3.35 (SD 0.47) points on a scale from 0 to 5. The highest-rated uMARS dimension was information (mean 3.88, SD 0.63), followed by functionality (mean 3.84, SD 0.87). Females reported a significantly higher uMARS total score than males (mean 3.47, SD 0.48 vs mean 3.23, SD 0.45; *P*=.03, Vargha and Delaney A [VDA] 0.66, 95% CI 0.53-0.77). The mean average of the NPS was 6.23 (SD 2.64) points (on a scale from 0 to 10), based on 43% (26/65 nonpromoters, 42% (25/65) indifferent, and 15% (9/65) promoters. A total of 7% (5/65) of those surveyed did not answer the question. When applying the NPS formula, the result is –26%. The NPS showed a positive correlation with the usage of mobile apps (*r*=0.39; *P*=.02). uMARS functionality was significantly higher rated by patients younger than 41 years (mean 4.17, SD 0.55 vs mean 3.54, SD 1; *P*<.001; VDA 0.69, 95% CI 0.56-0.80). Patients considering mobile apps as useful reported higher uMARS (*r*=0.38, *P*=.02). The uMARS app quality mean score was correlated with the frequency of using apps (*r*=–0.21, *P*<.001).

**Conclusions:**

The results revealed moderate acceptance and usability ratings, prompting further app improvement. Significant differences were observed between age and gender. Our results emphasize the need for further improvements in YogiTherapy.

## Introduction

Axial spondyloarthritis (AS) is an inflammatory rheumatic disease causing spine inflammation and chronic back pain [1,2]. This leads to stiffness and limited mobility due to deformity and ossification [3]. AS usually begins before age 45, impacting quality of life, daily activities, social participation, and employment [4-6]. Treatment combines pharmacological and nonpharmacological approaches [7,8] with physical therapy recommended by AS guidelines [9]. Intensive training can counteract spinal ankylosis and modulate inflammation positively [10]. The EULAR (European Alliance of Associations for Rheumatology) recommendation lists strength training and stretching as key activities for patients with arthritis [11]. General physical activity is crucial for long-term patient adherence and requires further research [12].

Digital Therapeutics (DTx), digital health applications (DHA), and telemedicine have gained traction [13], promoting active patient participation and adherence to therapy [14,15]. Interest in digitalization in rheumatology has grown, reinforced by the COVID-19 pandemic [16,17]. A survey identified DTx’s benefits as saving time (64%, 208/325), enabling health monitoring (48.9%, 159/325), and providing accurate information (40.9%, 133/325) [18]. High-quality apps for AS are limited worldwide. In an evaluation of 1253 English apps, only 2 were of high quality [19-21]. However, there is a demand for AS-specific apps, with high acceptance to use and pay for them [15,16,22].

In German-speaking countries, 5 fitness apps stand out: Assessment of SpondyloArthritis International Society (ASAS; Stichting Assessment of Spondyloarthritis International Society), Gymondo (Gymondo GmbH), Kaia Health (Kaia Health Software GmbH), Rheuma Auszeit (Deutsche Rheuma-Liga Bundesverband eV) and Axia (Applimeda). ASAS calculates disease activity scores and provides disease information [23]. Gymondo and Kaia Health offer fitness and pain management exercises but are not AS-specific [24,25]. Rheuma Auszeit includes mental and physical exercises but lacks disease progression tracking [26]. The Axia app, approved as a medical device, offers personalized training for patients with AS by tracking sports and physiotherapy sessions [27].

YogiTherapy (MaD Lab) is a complementary treatment for patients with rheumatism, combining yoga exercises and self-monitoring of disease progression [2]. It includes electronic patient-reported outcomes like The Bath Ankylosing Spondylitis Metrology Index (BASMI), The Bath Ankylosing Spondylitis Functional Index (BASFI), The Bath Ankylosing Spondylitis Disease Activity Index (BASDAI), and Ankylosing Spondylitis Quality of Life (ASQoL) [28], which can be shared with the physician. Yoga combines muscle strengthening and stretching, improving sleep, pain perception, mental health, and overall functionality [29-31]. Its adaptability makes it suitable for various fitness levels, including older adults or those with limited mobility [32]. Mind-body practices such as yoga, recognized by the WHO, are increasingly used for managing chronic rheumatic diseases, with evidence suggesting they improve patient-reported outcomes [33].

As Nitschke et al [34] and Truong et al [2] have already reported in detail on YogiTherapy, placing the app in the context of digitalization in rheumatology, we are adding our further results on the acceptance and usability of the app in a larger patient population here. Larger surveys are needed to ensure the app’s efficiency and security. We would like to take this opportunity to point out that further background information on YogiTherapy can be found in the previously published papers mentioned above. This study used the revised YogiTherapy app prototype. The objective is to assess the usability and acceptance of the YogiTherapy app in patients with AS, aiming for targeted adjustments to promote long-term use and optimize self-management.

## Methods

### Study Design

The study was carried out at the outpatient clinic of the Department of Rheumatology and Immunology at the University Hospital Erlangen in Germany. The participating patients were mainly recruited from May 2022 to June 2023. Subsequently, the data was analyzed. The expected publication was planned for 2024. Inclusion criteria were a diagnosis of AS according to the classification criteria of the ASAS [35] or axial involvement in the context of psoriatic arthritis [36]. All patients were invited to complete questionnaires on acceptance, usability, and disease-specific questions created with the SoSci Survey platform (SoSci Survey GmbH). Additional information on the patients’ demographic data, app affinity, physical activity, and laboratory data, such as C-reactive protein (CRP) and erythrocyte sedimentation rate (ESR), was collected.

### Application Description

The prototype of the YogiTherapy app was developed by engineering students from the Laboratory for Machine Learning and Data Analysis at Friedrich-Alexander-Universität Erlangen-Nürnberg (FAU) and doctors from Erlangen University Hospital. The app offers a training section with guided yoga videos. In the test section, patients can carry out disease-specific tests, the results of which are displayed graphically on a timeline. The app also provides information on the disease and dietary recommendations for rheumatic diseases.

In an initial survey [2], problems with design and app function were mentioned and revised [34].

**Figure 1:**
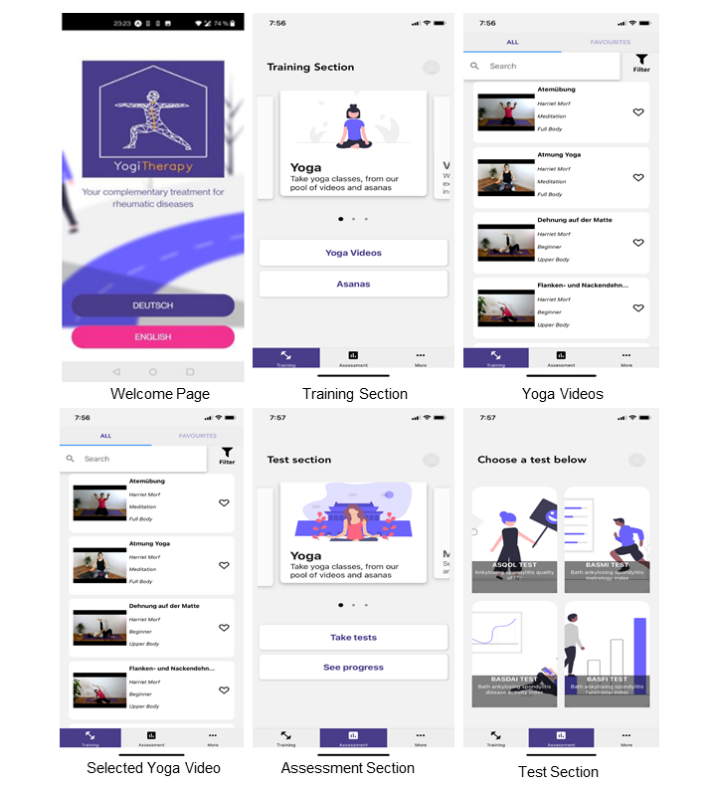
User interface and dashboard of the current version of the app YogiTherapy used in this study. Insight into the training area with exercise videos and into the test area to check progress on the reduction of axial spondyloarthritis–specific symptoms.

The functional requirements were retained, including a home page for language selection and registration (welcome page in Figure 1), a training area with yoga videos, with search, filter, and favorites options (training section, yoga videos, and “selected yoga video in Figure 1), an assessment area for completing disease-specific tests and tracking disease progression (assessment section and test section in Figure 1).

### Procedures

A medical doctor explained the functions of the app in person or over the phone. Patients were then able to briefly explore the app before completing the questionnaires. Participants rated the usability of the app using the German version of the User Version of the Mobile Application Rating Scale (uMARS) [37], a user-friendly version of the Mobile App Rating Scale [38] with 20 questions on engagement, functionality, aesthetics, information, and subjective quality on 5-point scales. Subjective quality is based on the willingness to recommend, the expected frequency of use, the willingness to pay, and the overall rating. The mean values of the subcategories result in the score, which ranges from 0 to 5 points.

The acceptance of YogiTherapy was measured using the net promoter score (NPS) [39]. Patients were asked on an 11-point Likert scale: “How likely is it that you would recommend this treatment to others?” Scores of 9 or 10 mean promoter, scores of 7 or 8 mean indifferent, and scores of 0 to 6 mean nonpromoter. This categorization predicts user behavior [39].

The patient survey also collected sociodemographic information, physical activity, and affinity for apps. Questions such as “How often do you use apps on a smartphone?” “Do you think the use of DTx is useful?” “Do you feel able to use DTx?” and “Do you use DTx?” were answered on a 5-point scale. Similar questions were used by Lambrecht et al [26] to investigate attitudes toward mHealth (mobile health). Physical activity was assessed using the BSA questionnaire, which records the frequency, duration, and type of activity over 4 weeks, in minutes per week [40]. In addition, BASDAI (0- to 10-point scale, where 0=low disease activity and 10=high disease activity), BASMI (0-10 point scale, where 0 is no limited and 10 is high limited mobility), and BASFI (0-10 point scale, where 0 means no limited and 10 means very limited function) scores were included in the analysis to define physical function, disease activity and mobility in patients with AS [28].

### Ethical Considerations

The study was approved by the institutional review board of the Medical Faculty of the University of Erlangen-Nuremberg, Germany (8_21_B, 26.02.2021). Participation in the survey was voluntary. All patients gave their written informed consent before study inclusion. All patients were coded with a consecutive number in a pseudonymization procedure. The data collected was stored and analyzed in a password-protected database. Only authorized persons had access to this data. Patients had the option of withdrawing their participation in the study at any time, whereby all personal data was irrevocably deleted. There was no financial compensation for participating in the study. The study was conducted in accordance with the ethical guidelines of the Declaration of Helsinki.

### Statistical Analysis

Descriptive analysis was performed. Correlation analysis was conducted, with Pearson correlation calculated for continuous variables, while polyserial correlation was determined for categorical variables. Differences in the median (for continuous variables) or the proportion (for categorical variables) between both sexes and 2 age categories were examined. The Wilcoxon Mann-Whitney rank sum test was used to test whether there were differences between the 2 groups regarding the median of each continuous variable. Due to skewed, nonnormal, or multimodal distributions, the outcomes were evaluated with nonparametric methods [41]. The Vargha and Delaney A (VDA) was used. VDA [42] suggested an effect size (ES) of 0.45-0.55 as a negligible effect, 0.56-0.63 (or 0.35-0.44) as a small effect, 0.64-0.70 (or 0.30-0.34) as a medium effect, and >0.70 (or <0.30) as a large effect. The chi-square test for count data was used to test whether there were proportion differences between the 2 groups for each categorical variable present in the data set. To measure the strength of the association between 2 categorical variables, Cramer V was computed, with a value of 1 corresponding to complete association and of 0 corresponding to no association between the variables. The type I error rate was set at α=.05. All statistical analyses were performed using R software (version 4.3.1, R Foundation for Statistical Computing) for Windows 10. The VDA and Cramer V were calculated using the effect size package [43]. Polyserial correlations were calculated using the psych package [44].

## Results

### Patient Characteristics

A total of 65 (100%) patients with AS participated in the study from May 2022 to June 2023, of whom 51% (33/65) were females. The mean age was 43.5 (SD 14.6) years. The mean CRP value was 1.63 (SD 3.69) mg/L, and the mean ESR value was 10.6 (SD 13.0) mm/hour. There were 48% (31/65) patients with a positive human leukocyte antigen B27 (HLA-B27) antigen test status. The BASDAI showed a mean average of 3.33 (SD 2.21) points. The BASFI was 2.12 (SD 1.87) points. The BASMI demonstrated 0.64 (SD 1.22) points.

In total, 52% (34/65) of patients reported using apps several times a day, and 57% (37/65) would be willing to use DTx regularly. When asked if they thought DTx was useful, 29% (19/65) answered “applies completely,” 45% (29/65) answered “applies,” and no participant rated the usefulness of DTx as “does not apply” or “does not apply at all.” The data is shown in Table S1 in Multimedia Appendix 1.

### YogiTherapy uMARS Rating

The usability of YogiTherapy was measured using the uMARS, as shown in Tables 1 and 2. The overall uMARS app quality mean score was 3.35 (SD 0.47; 0-5-point scale). Information received the highest score (mean 3.88, SD 0.63), followed by functionality (mean 3.84, SD 0.87), graphical design (mean 3.56, SD 1.26), and engagement (mean 3.40, SD 0.64). The uMARS app quality mean score was correlated with the frequency of using applications (*r*=–0.21, *P*<.001) and being a female (*P*=.03).

**Table 1:** User Version of the Mobile Application Rating Scale (uMARS) sections and mean scores by sex in a cross-sectional study of 65 patients with axial spondyloarthritis (51% female, 49% male) in Erlangen, Germany, from May 2022 to June 2023.

uMARS dimensions	uMARS mean score				*P* value
	Both sexes (n=65), mean (SD)	Female (n=33), mean (SD)	Male (n=32), mean (SD)		
Mean score	3.35 (0.47)	3.47 (0.48)	3.23 (0.45)	.04^a^	
Engagement	3.40 (0.64)	3.55 (0.66)	3.26 (0.59)	.07	
Functionality	3.84 (0.87)	3.93 (0.94)	3.76 (0.81)	.43	
Graphic design	3.56 (1.26)	3.59 (1.31)	3.52 (1.23)	.84	
Information	3.88 (0.63)	4.02 (0.54)	3.75 (0.71)	.09	
Subjective quality	3.15 (0.66)	3.28 (0.65)	3.02 (0.66)	.11	
App specific quality	3.26 (0.89)	3.43 (0.89)	3.08 (0.86)	.11	

^a^Significant at *P*<.05.

**Table 2:** User Version of the Mobile Application Rating Scale (uMARS) sections and mean scores by age in a cross-sectional study of 65 patients with axial spondyloarthritis (average age 43.3, SD 13.6 years) in Erlangen, Germany, from May 2022 to June 2023.

uMARS dimensions	uMARS mean score				*P* value
	Both age categories (n=65), mean (SD)	<41 years (n=31), mean (SD)	≥41 years (n=34), mean (SD)		
Mean score	3.35 (0.47)	3.39 (0.40)	3.32 (0.54)	.15	
Engagement	3.40 (0.64)	3.41 (0.60)	3.39 (0.68)	.38	
Functionality	3.84 (0.87)	4.17 (0.55)	3.54 (1.00)	<.001^a^	
Graphic design	3.56 (1.26)	3.72 (1.34)	3.41 (1.18)	.20	
Information	3.88 (0.63)	3.93 (0.61)	3.84 (0.67)	.09	
Subjective quality	3.15 (0.66)	3.15 (0.56)	3.16 (0.75)	.93	
App specific quality	3.26 (0.89)	3.07 (0.89)	3.42 (0.86)	.40	

^a^Significant at *P*<.05.

### NPS Rating

**Figure 2:**
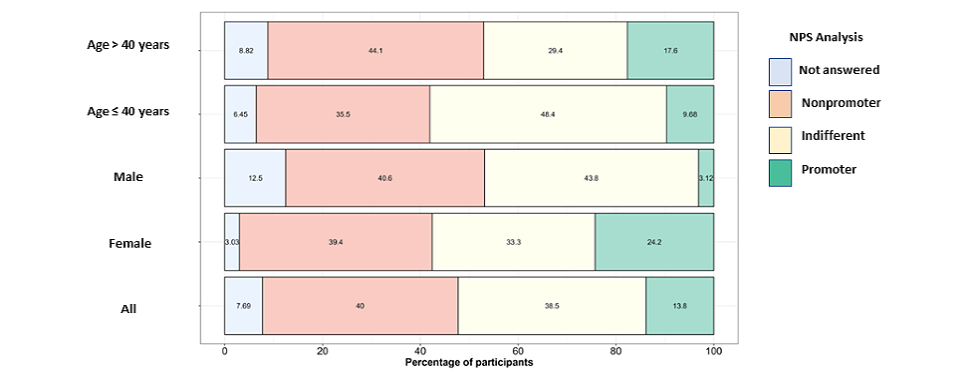
Bar chart of net promoter score (NPS) analysis showing the percentage of participants classified as promoter, indifferent, or nonpromoter of YogiTherapy by age and sex. This cross-sectional study included 65 patients with axial spondyloarthritis (51% female, 49% male) in Erlangen, Germany, from May 2022 to June 2023.

When analyzing the NPS, the participants were divided into 43% (26/65 nonpromoters, 42% (25/65) indifferent, and 15% (9/65) promoters, as shown in Figure 2. The mean score was 6.23 (SD 2.64). There were more females in the promoters (25%, 8/9). There was a positive correlation with the app quality mean score (*r*=0.64; *P*<.001), the uMARS subjective quality score (*r*=0.75; *P*<.001), uMARS engagement score (*r*=0.53; *P*<.001), uMARS information score (*r*=0.43; *P*<.001) and the uMARS functionality score (*r*=0.31; *P*<.001).

Patients who used DTx definitely demonstrated higher NPS ratings (*r*=0.39; *P*=.02). Compared with males, more females chose “applies completely” for both the usage of DTx (Cramer V=0.36*; P*=.01) and DTx making sense questions (Cramer V=0.38; *P*=.01).

When applying the NPS formula, as a subtraction of the percentage of nonpromoters from the percentage of promoters, the result is –26%.

When examining sex differences, it was noticeable that females had a higher ESR (mean 12.5, SD 12 mm/h vs mean 8.31, SD 14 mm/h; *P≤*.001) and a higher uMARS app quality mean score than males (3.47, SD 0.48 vs 3.23, SD 0.45; *P*=.03).

There were several differences between the 2 age categories, with younger participants (<41 years) that had a lower ESR (mean 6.16, SD 4.84 mm/h; *P*<.001), lower CRP (mean 0.77, SD 2.27 mg/L; *P*=.12), lower BASMI (0.21, SD 0.42; *P*=.03), and higher uMARS functionality scores (mean 4.17, SD 0.55; *P*<.001) than participants 41 years or older (ESR: mean 15.9, SD 17.2 mm/h; CRP: mean 2.60, SD 4.67 mg/L; BASMI: mean 1.12, SD 1.62; uMARS functionality score: mean 3.54, SD 1.00).

The uMARS functionality score (*r*=–0.35, *P*<.001) and the uMARS graphic design score (*r*=0.13, *P*<.001) were all correlated with the frequency of DTx use, as was the uMARS app quality mean score (*r*=–0.21, *P*<.001). The uMARS functionality score also positively correlated with the frequent usage of DTx several times a day (*r*=0.48, *P*<.001).

Patients who applied completely to the question of DTx making sense were positively correlated with the total uMARS (*r*=0.38, *P*=.02). The question about the use of DTx was positively related to the uMARS subjective quality (*r*=0.34, *P*=.03).

The evaluation of the participants’ sports activity reported in the BSA questionnaire showed that the younger the patients were, the higher the level of sports activity reported (*r*=–0.37, *P*=.55).

## Discussion

### Principal Findings

The aim of this study was to assess the usability and acceptance of the YogiTherapy app to provide complementary exercise therapy for patients with AS. Overall, the quality of the app was good, with the functionality section rated particularly highly, whereby the acceptance was rather average.

The quality of the app YogiTherapy was rated as “mediocre,” as indicated by a score in the middle range of the uMARS. The information section of the uMARS received the best rating, followed by the functionality section with the second-highest value (mean 3.84, SD 0.87). This is consistent with the findings of a systematic review of 18 pain-related mobile apps also using the MARS score, where the best ratings were related to technical aspects of the app itself, such as “functionality” [45]. Furthermore, the information aspect was low here, which was explained by the fact that most apps are commercial, and a few are developed by scientific institutions. Thus, YogiTherapy has an advantage as a DTx developed by a university hospital [2,34]. The widest range of uMARS scores was found in the subjective quality of the app, engagement, and aesthetics, which is why these aspects should be improved [45].

With an overall mean score of 3.35 (SD 0.47) on a scale of 5 points in uMARS, the quality rating is slightly lower than the rating of 9 other mobile apps for patients with rheumatic diseases, which ranged from 3.44 to 4.19 [46]. “Rheuma Auszeit” achieved the highest uMARS score. “Rheuma Auszeit” was the only 1 of the 9 apps to offer instructions for mental and physical exercises. However, “Rheuma Auszeit” did not have a feature to track the disease progression. In comparison, the Axia app would be a possible alternative to exercise therapy for patients with AS, as initial studies have already indicated.

An interesting concept from the developers is the possibility of combining exercises from the app with everyday tasks. This should probably strengthen adherence in the long term and improve movement competence, an advantage that Azhar and Dhillon [47] also identified in their review. In their systematic review to identify factors influencing the effective use of mHealth apps for self-care, the most commonly identified factors were perceived usefulness, perceived ease of use, behavioral intention to use, social influence, and self-efficacy [47]. The app Axia offers users over 250 exercise videos for personalized therapy, special programs for acute pain and intensive training, a knowledge library with 56 interactive articles on AS management, symptom tracking, a progress dashboard, relaxation exercises, and medication monitoring [48]. As the approach to supporting patients is similar to that of YogiTherapy, we could imagine that our app would be particularly useful for patients who would like to practice yoga in addition to a physiotherapy approach. Even though YogiTherapy scored lower than Axia or Rheuma Auszeit in the uMARS, YogiTherapy provides holistic support to users [34].

In a recent survey on the need for an app in AS, the willingness to use an app appears to be greatest in patients <60 years of age, patients undergoing biologic therapy, and patients with frequent back pain [49]. We divided our age categories into patients <41 years and ≥41 years based on the median age. The younger age category achieved a slightly higher value in the uMARS app quality mean score (3.39, SD 0.40) than the older age category (3.32, SD 0.54; *P*=.78). In the case of functionality, younger patients scored the app significantly higher than older patients (4.17, SD 0.55 vs 3.54, SD 1; *P*<.001). However, the patients ≥41 years achieved a higher mean score in the section of app specific quality (3.07, SD 0.89 vs 3.42, SD 0.86; *P*=.08). In a recent study on online physiotherapy in patients with AS, Paul et al [19] also showed a positive effect of the online program on disease parameters such as BASDAI, BASMI, and AsQoL. The statement that patients with higher disease activity indicated a greater need for an app was only partially reflected in our data. There was just 1 significant correlation between uMARS functionality and higher CRP (*r*=0.02; *P*=.03). As females in particular rated the app well, it was also noticed here that ESR was significantly higher than in male patients (12.5, SD 12 vs 8.31, SD 14; *P*<.001). In our study, older patients showed values of a greater inflammatory process and poorer functionality; yet, the app was rated slightly lower in quality. This result could be falsified by the different age categories selected. Interestingly, however, there were no differences in perceived disease activity depending on sex and age.

Age-related differences were noted, but these could not be recorded more precisely as experience using the app was not assessed [34].

The uMARS app quality mean score was correlated with the frequency of using DTx (*r*=–0.21; *P*<.001). Looking at the correlations between the individual uMARS sections and the stated frequency of DTx use, the app affinity, it is noticeable that the correlation *r* is only positive (as a higher point value in the frequency question represents a rarer frequency) in the Graphic Design section (*r*=0.13; *P*<.001). This could indicate that YogiTherapy is particularly suitable for newcomers to app use. Patients who used DTx demonstrated higher NPS ratings (*r*=0.39; *P*=.02). Females rated the usefulness of DTx significantly higher, 46.9% (15/19) than men, 12.1% (4/19). This observation was also evident in the overall uMARS score (3.47, SD 0.48 vs 3.23, SD 0.45; *P*=.03) and in the NPS, where the number of promoters among females was 8 (8/9, 25%) and 1 (1/9, 3%) among males. It is known that patients who are aware of their self-efficacy in relation to the treatment of their chronic disease show better compliance with physical exercise [50]. In addition, a positive correlation between medication self-efficacy and adherence to treatment was found in a systematic study of the relationship between self-efficacy, health control beliefs, and adherence to treatment [51]. Unfortunately, we do not have information about patients’ medication. Patients are looking for alternative treatments to improve their symptoms. These patients may be more open to exploring different treatment options and innovative tools such as DTx, which can help to improve care and support. This was also observed in a study of “the AxSpA Live app” [52]. The app offers a diary function in which various disease variables can be documented. Their results showed that older patients with more disease activity used the app more often.

The NPS showed a less positive result with a mean value of 6.23 (SD 2.64, range 0-10) and a result of –26% in the NPS formula. This could indicate that patients would probably not actively recommend the app to others. The discrepancy between the NPS and the overall satisfaction rating was also reported by Seppen et al [53]. These authors cited possible cultural differences in the type of rating as the reason for this; European people would tend to give fewer extreme ratings compared with Americans, and the NPS originated in the United States [54]. This rather poor result in the NPS score gives us the motivation to continue to improve YogiTherapy in terms of usability and acceptance.

Our evaluation of YogiTherapy focused on the impact on users’ health behaviors, which were assessed in the “App-specific quality” section of the uMARS. Patients rated statements such as “The app increases awareness of the importance of addressing health behaviors” and “The app encourages intention and motivation to improve health behaviors” on a 5-point scale. The mean score in this category was 3.26 (SD 0.89), with women (mean 3.43, SD 0.89) and participants aged 41 years and older (mean 3.42, SD 0.86) scoring higher than men (mean 3.08, SD 0.86) and those aged 41 years and younger (mean 3.07, SD 0.89). This suggests that female and older users may better recognize the value of health apps. Women may especially be more open to using DTx as they are more mindful of their health or already have experience using health services [55]. Younger users may have different priorities, as our data show a negative correlation between age and physical activity (*r*=–0.37; *P*=.55), suggesting that younger patients lead a more active lifestyle. In addition, younger patients had lower ESR values (mean 9.33, SD 5.69 vs mean 19.0, SD 18.9; *P*=.002) and better physical activity levels (mean 0.50, SD 0.71 vs mean 2.50, SD 1.87; *P*=.03) and disease-specific physical function (mean 1.85, SD 1.53 vs mean 2.81, SD 2.28; *P*=.05) than older patients. This statement is also supported by the fact that in our collected data, younger patients had better values for range of motion (mean 0.50, SD 0.71 vs mean 2.50, SD 1.87; *P*=.03) than older patients.

The influence of DTx on health behavior has already been investigated. The chances of behavioral change were low to moderate on average (App Behavior Change Scale [ABACUS] score: mean 8.07, SD 2.30) in a sample of 60 apps [56]. ABACUS is a validated and objective tool that uses 21 items categorized into knowledge and information, goals and planning, feedback and monitoring, and actions. The reachable score lies between 0 and 21. The higher the score, the higher the potential for promoting behavior change [57]. It was found that apps for patients with multimorbidity tended to have a higher overall ABACUS score. The most common features in these apps that supported behavior change were self-monitoring of physiological parameters such as blood pressure (38/60, 63% apps), weight and diet (25/60, 42% apps), physical activity (22/60, 37% apps), and stress management (22/60 apps, 37%) [56]. YogiTherapy, therefore, has the potential to support behavioral change through its functions.

As many study participants were given information about the app over the phone, contacting them became more impersonal, which could have had a negative impact on their evaluation of the app. Therefore, potential users should be introduced by trained staff members in one-to-one contact. Practicing and training with an app is not suitable for every patient, and a doctor consultation should take place beforehand. Older patients (≥41 years) expressed concern in verbal feedback that yoga was too demanding for their previous yoga experience and their physical fitness. The “RheumaAuszeit” app, which contains relaxation exercises, received a higher uMARS score [26,46]. These findings allow consideration of offering relaxation exercises in the YogiTherapy app as well. Although the app already provides precise instructions on alternative exercises and postures, it seems sensible to offer an analog introduction to the sport by trained staff members, especially for newcomers to yoga practice.

In addition, we analyzed the responses of 45 other patients who watched and performed the freely available yoga videos used in the YogiTherapy app. At the end of each video, a survey appeared asking about the difficulty level of the yoga exercises and whether they would recommend the videos to a friend. The second question matched the NPS question and was therefore also asked of the 65 patients who took part in our survey. As expected, we found a higher score (mean 8.69, SD 1.97) for recommending the videos to friends among the study participants who, unlike the other patients, had already engaged with the videos over a longer period of time. There may be a bias here, as the 45 patients were not part of the study.

Our study has limitations, starting with the study design, which aimed to evaluate the app based on first impressions. However, in order to evaluate the true benefit of the YogiTherapy app on patients with AS, further data over a longer period of time compared with standard physiotherapy needs to be collected.

### Conclusion

In conclusion, our study assessed the usability and acceptance of the YogiTherapy app in patients with AS. The present findings highlighted potential age and gender-related variations. Our results emphasize the need for further improvements in DTx for rheumatology care.

## Appendix

Multimedia Appendix 1 Patients' demographic characteristics (n=65).

## Abbreviations

ABACUS: App Behavior Change Scale
ANR: French National Research Agency
AS: axial spondyloarthritis
ASAS: Assessment of SpondyloArthritis International Society
ASQoL: Ankylosing Spondylitis Quality of Life
BASDAI: Bath Ankylosing Spondylitis Disease Activity Index
BASFI: Bath Ankylosing Spondylitis Functional Index
BASMI: Bath Ankylosing Spondylitis Metrology Index
CRP: C-reactive protein
DHA: digital health application
DTx: digital therapeutics
ES: effect size
ESR: erythrocyte sedimentation rate
EULAR: European Alliance of Associations for Rheumatology
FAU: Friedrich-Alexander-University
HLA-B27: human leukocyte antigen B27
mHealth: mobile health
NPS: net promoter scale
uMARS: User Version of the Mobile Application Rating Scale
VDA: Vargha and Delaney A

## Data Availability

The data sets generated and analyzed during this study are available from the corresponding author upon reasonable request. The majority of the data is included in this published article.
