# Supplementary material for: Complementary App-Based Yoga Home Exercise Therapy for Patients With Axial Spondyloarthritis: Usability Study
Source: JMIR Form Res. 2024 Sep 19;8:e57185. doi: 10.2196/57185 (PMC11450357; doi:10.2196/57185)
Supplement: Multimedia Appendix 1 [file formative_v8i1e57185_app1.docx]

**Table S1**.

| **Characteristics** | **Entire population (n=65)** | **Female (n=33)** | **Male (n=32)** | **Age < 41 years (n=31)** | **Age ≥ 41 years (n=34)** |  |
| --- | --- | --- | --- | --- | --- | --- |
| **Usage of health applications,**  **n (%)** | |  |  |  |  |  |
| No | | 28 (43) | 10 (31) | 18 (55) | 13 (42) | 15 (44) |
| Yes | | 37 (57) | 23 (72) | 14 (42) | 18 (58) | 19 (56) |
| **Frequency usage of applications score, n (%)** | |  |  |  |  |  |
| Several times a day | | 34 (52) | 15 (47) | 19 (58) | 18 (58) | 16 (47) |
| At least 1x a day | | 17 (26) | 9 (28) | 8 (24) | 6 (19) | 11 (32) |
| At least 1x a week | | 8 (12) | 5 (16) | 3 (9) | 5 (16) | 3 (9) |
| Seldom | | 5 (8) | 3 (9) | 2 (6) | 2 (6) | 3 (9) |
| Never | | 1 (2) | 1 (3) | 0 | 0 | 1 (3) |
| **Health applications making sense score, n (%)** | |  |  |  |  |  |
| Does not apply at all | | 0 | 0 | 0 | 0 | 0 |
| Does not apply | | 0 | 0 | 0 | 0 | 0 |
| Neutral | | 17 (26) | 8 (25) | 9 (27) | 9 (29) | 8 (24) |
| Applies | | 29 (45) | 10 (31) | 19 (58) | 12 (39) | 17 (50) |
| Applies completely | | 19 (29) | 15 (47) | 4 (12) | 10 (32) | 9 (27) |
